# New Conjugates of Quinoxaline as Potent Antitubercular and Antibacterial Agents

**DOI:** 10.1155/2016/6471352

**Published:** 2016-03-08

**Authors:** Ramalingam Peraman, Rajendran Kuppusamy, Sunil Kumar Killi, Y. Padmanabha Reddy

**Affiliations:** ^1^Raghavendra Institute of Pharmaceutical Education and Research (RIPER), Andhra Pradesh 5215002, India; ^2^College of Pharmacy, Gulf Medical University, Ajman, UAE; ^3^Medicinal Chemistry Division, National Institute of Pharmaceutical Education and Research (NIPER), Kolkata 700032, India

## Abstract

Considering quinoxaline as a privileged structure for the design of potent intercalating agents, some new sugar conjugates of quinoxaline were synthesized and characterized by IR, ^1^HNMR, ^13^C NMR, and mass spectral data.* In vitro* testing for antitubercular and antimicrobial activities was performed against* Mycobacterium tuberculosis H*_*37*_ *Rv* and some pathogenic bacteria. Results revealed that conjugate containing ribose moiety demonstrated the most promising activity against* Mycobacteria* and bacteria with minimum inhibitory concentrations (MIC) of 0.65 and 2.07 *μ*M, respectively. Other conjugates from xylose, glucose, and mannose were moderately active whilst disaccharides conjugates were found to be less active.* In silico* docking analysis of prototype compound revealed that ATP site of DNA* gyrase* B subunit could be a possible site for inhibitory action of these synthesized compounds.

## 1. Introduction

The World Health Organization (WHO) tuberculosis report 2015 stated that an annual total of new tuberculosis (TB) cases, which had been about 5.7 million until 2013, rose to more than 6 million in 2014 (an increase of 6%). Unfortunately, extensively drug-resistant TB (XDR-TB) cases had been reported by 105 countries in the year 2015 and among them 9.7% of patients with multidrug resistant-TB (MDR-TB) have recorded positive for XDR-TB [1]. These reports indicated that the prevalence of the disease is the worst and is due to the emergence of multidrug resistant strains of* Mycobacterium tuberculosis H*_*37*_ *Rv* [2, 3]. Thus, these resistant states of tuberculosis (MDR-TB and XDR-TB) have increased the concern about tuberculosis as an incurable disease and as a threat to public health [4, 5]. Recently, World Health Organization (WHO) has advised against the strategic therapy by adding a new drug into a failing antitubercular drug regimen that increases the drug resistant behavior of the strain. It indicated that the present treatment is extremely complicated and needs new drug with novel mechanism exclusively to fight against multidrug resistant strains. To add, the immune compromised patients with the longer durations of therapy also emphasize the need for new antitubercular molecule with novel mode of action [6–8].

In search of heterocyclic moiety for the design of new antitubercular agent, quinoxaline is well known as privileged structure (PS) for easy construction of chemical scaffold [9, 10]. Quinoxaline class of drugs (triostin A, echinomycin) are well documented for their biological accessibility and they were used as DNA intercalating agents [11]. In the light of literature, several reports on biologically active quinoxaline derivatives such as tricyclic quinoxaline, pyridinylquinoxalines, and pyridinylpyridopyrazines (as potent inhibitors of protein kinase) [12], olaquindox, quindoxin, and carbadox (as potent antibacterial agents) [13], and quinoxaline-2-carboxylate 1,4-dioxide (as potent antitubercular agents) [14] have been reported. Outstandingly, few derivatives such as, 1,4-dioxides, sulphonamide, spiro, schiff bases, mannich bases, keto, and amido are reported to possess potent antimicrobial property. Among them, spiro [15], 1,4-dioxide [16], and schiff base [17] of quinoxaline are proven to be the most suitable antibacterial and antitubercular leads. Nonetheless, the reliability of above agents for new drug design is not optimistic because of their structural toxicity contributed by high lipophilicity and resistance to metabolism.

With reference to the biological safety profile of sugar conjugates derived from natural and synthetic agents [18–20], and in continuation with our earlier studies on quinoxaline [21–25], herein we report some saccharide (sugar) linked quinoxaline. In this work, the hydrazino intermediate was preferred to achieve sugar conjugates in order to mimic the structural resemblance to nucleosides and other active hydrazide drugs. In synthetic part, quinoxaline 2,3 (1H, 4H)-diones (**1**) was used to prepare hydrazino quinoxaline as an intermediate (**2**); then the intermediate (**2**) was subsequently condensed with different sugars (aldohexoses, aldopentoses and disaccharides) to afforded sugar conjugates of quinoxaline (**3a–f**). Compounds were purified by column chromatography and the purity was confirmed using TLC and HPLC technique. The structure establishment was done by IR, ^1^HNMR, and ^13^C NMR and mass spectral data. All synthons including starting compounds were screened for their antitubercular (*Mycobacterium tuberculosis H*_*37*_ *Rv)* and antibacterial (*Staphylococcus aureus, Escherichia coli, Proteus vulgaris, Pseudomonas aeroginosa*, and* Salmonella typhi*) activities. Results were obtained as minimum inhibitory concentration (MIC) in micro molar units (*μ*M). As an attempt to predict the mode of action of these compounds, a prototype compound was evaluated on DNA gyrase by* in silico* docking analysis.

## 2. Results and Discussion

### 2.1. Chemistry

**Figure 1:**
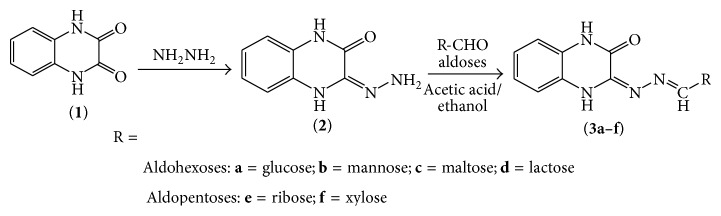
Schematic route for synthesis of sugar conjugates of quinoxaline.

**Figure 2:**
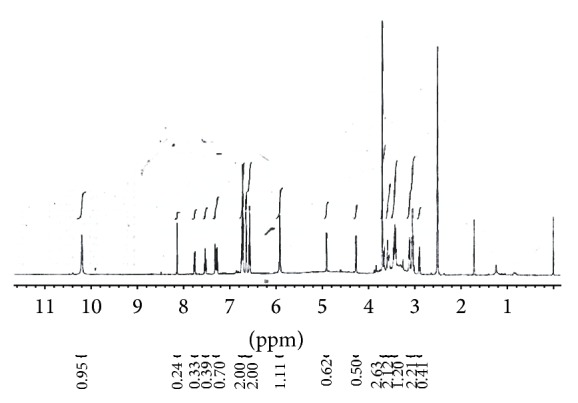
H^1^- NMR spectrum of 3-(2-*β*D-glucopyranosylidene hydrazinyl)quinoxalin-2(1H)-one (**3a**).

The synthetic route for sugar conjugates was designed in two step reactions (Figure 1). The starting material quinoxaline 2,3(1H, 4H)-dione** 1** was obtained as yellowish crystals from an acid catalyzed condensation reaction between* ortho* phenylenediamine and oxalic acid. Chemical reactions of synthetic scheme were performed based on conventional heating method. Reaction progress was monitored on silica gel coated TLC plate. The temperature of the reaction for converting compound** 2** to** 3a–f** was controlled, because of thermolabile nature of sugars that are vulnerable for charring at higher temperature. Compounds were found to be stable crystals except compound** 2**. The % yield was more than 60 except for disaccharide derivatives (**3c** and** 3d**). All monosaccharide derivatives were yellowish crystals whilst disaccharides derivatives appeared as creamy white. In TLC, the retardation factors (*R*_*f*_ value) for sugar conjugates (**3a–f**) were lower than compounds** 1** and** 2**, thus indicating the more polar nature of sugar conjugates. The calculated log⁡*P* and Clog⁡*P* values of sugar conjugates are in good agreement with our prediction that they are more hydrophilic than parent compound (**1**). The melting point of the synthesized compounds was more than 200°C; this could be due to the high degree of hydrogen bonding offered by –OH residues of sugar moiety. The physical data of the synthesized compounds are shown in Table 1. Structure of the synthesized compounds was established by UV, IR, ^1^H NMR, ^13^C NMR, and mass spectral data. Assignment of proton and carbon to the structure was performed based on the report by Bailey and Butterfield [26]. The bathochromic shift of *λ*_max_ for compound** 3a–f** indicated the formation conjugated C=N bond. The conversion of quinoxaline-2,3(1H, 4H)-diones** 1** to intermediate** 2** was established by spectral characteristics such as (a) disappearance of coupled vibration of C=O, (b) appearance of coupled band for NH_2_, (c) the appearance of ^13^C NMR signal for C=N, and (d) ^1^H NMR for –NH_2_. The conjugate formation with sugar was established by (a) ^1^H NMR signal at 4.23 and 4.97 ppm for anomeric CH– of sugar, (b) ^13^C NMR signals within 100–60 ppm for sugar structure residue, and (c) the presence of broad absorption band for –OH group in IR spectrum (3400–3600 cm^−1^). The detailed spectral characteristics of the compounds are shown in Table 2. A prototype ^1^H NMR spectrum of glucose conjugate is shown in Figure 2.

### 2.2. Antibacterial and Antitubercular Activities

Compounds (**1** to** 3a–f**) were screened for their* in vitro* antibacterial and antitubercular activities using microdilution assay method and microplate alamar blue assay (MABA) method, respectively. The obtained results were presented in Table 3. The concentration range used for screening was from 0.04 to 9.6 *μ*g/mL, and then the obtained MIC (in *μ*g/mL) was converted to micromolar (*μ*M) concentration. Result revealed that compound** 3e** (ribose conjugate) exhibited promising antibacterial and antitubercular activities with respective MIC of 2.07 and 0.65 *μ*M. Compound** 3f** (xylose conjugate) was also equally effective against* S. aureus* and* E. coli*, but not against other pathogens of this study. Antibacterial activity of compound** 3a** (glucose conjugate) was quite considerable (MIC: 14.2 *μ*M), but it was equipotent to compound** 2**. Among aldohexoses, compound** 3a** (glucose) was moderately active against* M. tuberculosis* (MIC: 7.1 *μ*M) than compounds** 1** and** 2**. It was noted that there was no true compound that was active against* Salmonella typhi*, except compound** 3e** (MIC: 7.8 *μ*M). It was evident that conjugates of pentoses (compounds** 3e** and** 3f**) showed promising antibacterial activity against* Staphylococcus aureus* and* Proteus vulgaris* and are quite comparable to ciprofloxacin. The intermediate (compound** 2**) showed MIC of 3.6 *μ*M against* E. coli*, as similar to conjugates of pentoses (**3e** and** 3f**); thus the potency of compound** 2** is in good agreement with our earlier studies.

**Figure 3:**
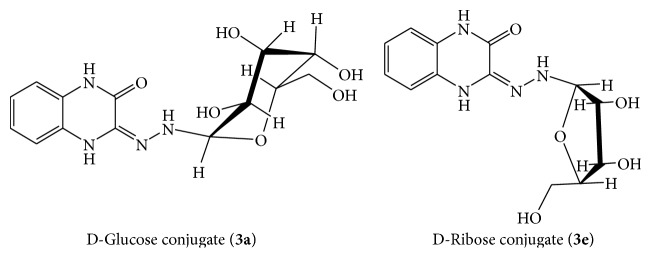
Structures of most active compounds: 3-(2-*β*D-glucopyranosylidene hydrazinyl)quinoxalin-2(1H)-one (**3a**) and 3-(2- *α* D ribofuranosylidene hydrazinyl)quinoxalin-2(1H)-one (**3e**).

The screening for antitubercular activity revealed that all sugar conjugates exhibited better activity as compared to parent compound** 1** and intermediate compounds** 2**; thus clubbing of sugar to quinoxaline could be a beneficial approach in design of antitubercular agents. Among them, conjugates monosaccharide showed better activity as compared to the hydrazine derivative** 2** and disaccharides (**3c** and** 3d**). The most promising antibacterial activity was shown by ribose conjugate** 3e** (MIC: 0.65 *μ*M), followed by xylose** 3f**, glucose** 3a,** and mannose** 3b** conjugates with respective MIC values of 3.9, 7.1, and 7.1 *μ*M. Overall, it was noticed that disaccharides linkage has low value in design of antibacterial and antitubercular leads as compared to monosaccharides. Among monosaccharides, aldopentose derivatives are more potent as compared to aldohexoses. The structures of active compounds are shown in Figure 3. The effect of stereoisomerism of sugar in this study was quite significant and this can be supported by the comparison between ribose derivative (**3e**) and xylose derivative (**3f**). It was noted that ribose derivative is 7 times more potent than xylose derivative. However, both compounds** 3a** and** 3b** are equipotent in antitubercular activity but differ in antibacterial activity.

In view to antibacterial screening results,* Salmonella* species was resistant to all compounds except compounds** 3e** and** 3f**, which indicated the importance of aldopentose in design of antibacterial leads against* Salmonella* species. Antitubercular activity of all conjugates (log⁡*P* > −1) is better than compounds** 1** and** 2** (log⁡*P* < 1), which indicated the importance of log⁡*P* parameter in defining the activity. The antitubercular activity of disaccharide conjugates** 3c** and** 3d** (maltose and lactose conjugates) is relatively very low (15 times less than ribose) and the possible reason could be that they are high molecular weight compounds with more hydrophilic (log⁡*P* more than −3.0) nature. Thus, disaccharide linked conjugates are likely to exhibit less permeability to cell wall; thereby it leads to inadequate cellular concentration. Aldopentose accounts for their better penetration into the cell wall of* Mycobacterium* species conjugates (**3e** and** 3f**) because of their low molecular weight and optimal hydrophilicity (log⁡*P* > −1 and <−2). In addition, stereoisomer effect also plays important role in their relative potency.

### 2.3. *In Silico* Docking Analysis

**Figure 4:**
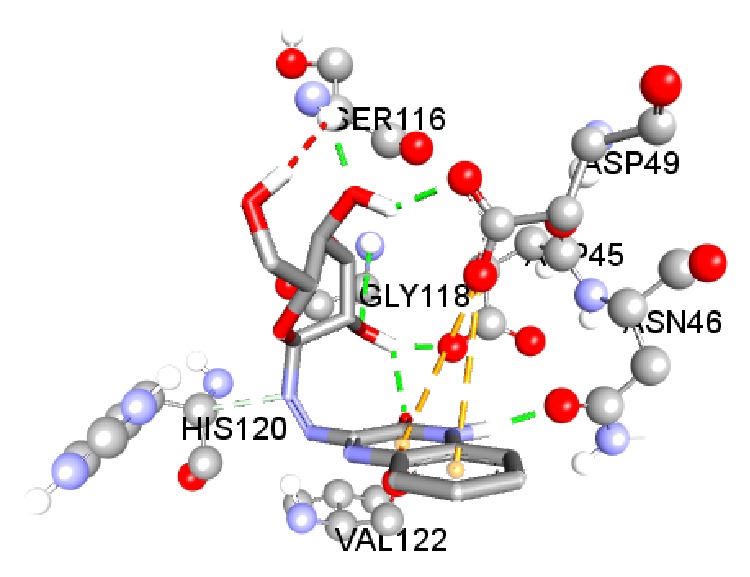
Key molecular interactions (hydrogen bonds (green), electrostatic interactions (orange)) of compound** 3a** with the key amino acids present in ATP binding pocket of DNA gyrase enzyme. (PDB ID 4HYP).

The DNA gyrase is an enzyme, responsible for the topological state of DNA and playing important role in transcription process. It is the target for few antibiotics such as many antibiotics, including nalidixic acid, novobiocin, and ciprofloxacin. Particularly, fluoroquinolones act as an antibacterial through DNA cleavage by inhibiting ATPase activity of DNA gyrase. As Adenosine itself contains a nucleotide and a sugar moiety, we predicted that the synthesized molecules (**3a–f**) may inhibit the ATPase binding site of DNA gyrase. Further to elucidate the probable mechanism of action of our designed compounds, we have initially done the molecular docking studies of our designed molecule (**3a**) with DNA gyrase B subunit using PDB ID 4HYP. Results shown that the structure of compound** 3a** tightly bounds to the ATP binding site (Figure 4) and showed binding energy of −7.0. The hydroxyl groups (–OH) of sugar moiety showed hydrogen bonds with SER 116, GLY 118, Asp 45, ASN46, ASP 49, and VAL 122, whilst the quinoxaline moiety accounted for pi-pi- stacking interaction with ASP 49 and Asp 45. The linker imine group is well recognized for nonclassical hydrogen bonding interaction with the HIS120, PHE 104. Recent reports say that the hydrogen bonding interactions ASN46, ASN45, and GLY118 are crucial for the activity. Thus, the molecular docking results suggested that the synthesized compounds may inhibit the ATPase activity of DNA* gyrase* by firmly binding into the ATP binding pocket. The docked molecule with DNA* gyrase* is shown in Figure 4.

## 3. Experimental

### 3.1. Materials

Purity of compounds was confirmed using TLC (aluminum 60F_254_) and HPLC (Agilent LC 1200, C_18_ (250 mm × 4.6 mm, 5 micron) Column) techniques. The melting point was determined by open capillary tube method and is uncorrected. The UV spectrum was obtained using a UV-Visible spectrum (Shimadzu UV-Visible Double beam Spectrometer). Functional groups were established by IR spectra using a Fourier transform-infrared spectrophotometer (Alpha E, Bruker, ATR technique) at resolution of 4 cm^−1^. The carbon and proton of the structure components were assigned using proton nuclear magnetic resonance spectrum (^1^H NMR, 400 MHz) using a Varian VXR Unity (California) using TMS as internal standard and DMSO-d_6_/CDCl_3_ as solvent and carbon nuclear magnetic resonance spectrum (^13^C NMR, 300 MHz) using Brukers UXNMR (Germany) using DMSO-d_6_/CDCl_3_ as solvent. The mass spectrum from Shimadzu LCMS -2010A (Japan) was used to confirm molecular weight. All reagents used were of analytical or synthetic grade.

### 3.2. Synthesis and Characterization

The starting material, quinoxalin-2,3-dione 1, was synthesized as stated in literature [27]. The product was obtained as yellowish silky needle crystals. The conversion of compound 1 to compound 2 was carried out as per our earlier report [28].

#### 3.2.1. Synthesis of 3-(2-Hexopyranosylidene hydrazinyl)quinoxalin-2(1H)-one (**3a–d**) and 3-(2-Pentofuranosylidene hydrazinyl)quinoxalin-2(1H)-one (**3e**,** 3f**)

A mixture of compound 2 (0.015 mol) and various hexoses and riboses (0.015 mol) in absolute alcohol containing catalytic amount of acetic acid was heated under reflux for 8 h. Care was taken to avoid charring of carbohydrates; if so reaction mixture was discarded. The reaction mixture was cooled and allowed overnight in refrigerator. The formed product was filtered and washed with small quantity of purified water and recrystallized from 95% ethanol. The purity of the product was confirmed on silica gel coated TLC plate and HPLC analysis.

All compounds were subjected for purification by column chromatography. The obtained compounds purity was tested using HPLC technique. Thus obtained compounds were characterized by UV, IR, ^1^H NMR, and ^13^C NMR and mass spectral data. The physical and spectral characteristics of the synthesized compounds are presented in Tables 1 and 2, respectively.

### 3.3. Antibacterial and Antitubercular Activities

#### 3.3.1. *In Vitro* Antibacterial Screening by Microdilution Method

Compounds were tested against bacterial pathogens, namely,* Staphylococcus aureus ATCC 25923*,* Escherichia coli ATCC 25922*,* Proteus vulgaris ATCC 29905*,* Pseudomonas aeroginosa ATCC 27853,* and* Salmonella typhi ATCC 14028* by broth microdilution technique using 96-well plate. The plate included positive control, negative control, blank and serial dilution tests, and standards (in DMSO) in the concentration range from 0.04 to 9.6 *μ*g/mL. After the addition of 100 *μ*L inoculums (2 × 10^6^ test organisms/mL) to wells, the plates were incubated at 36 ± 1°C. After 18 hours of incubation, the final volume of each well was made up to 200 *μ*L and then 20 *μ*L of 10-fold dilution of Alamar blue was added. Then plate was incubated for 30 min. The color intensity of each well was documented, and the MIC was recorded. The lowest concentration used that did not result in the change of colour from blue to pink. The results were calculated in *μ*M concentrations and shown in Table 3.

#### 3.3.2. Antitubercular Screening by Microplate Alamar Blue Assay (MABA) Method

A stock solution of the test compounds was prepared in DMSO at 1 mg/mL and was sterilized by passage through 0.45 *μ* Nylon based membrane filters. Controls received 50 *μ*L DMSO whilst isoniazid (INH) was included as positive drug control. The inoculum used was 1 : 100 dilutions to represent 1% of the mycobacterial population (10^2^–10^3^ CFU/mL). All the compounds were screened for antitubercular activity by serial addition at a concentration range from 0.04 to 9.6 *μ*g/mL. At the end of 5th day of incubation, 50 *μ*L of 1 : 1 dilution of Alamar blue stock solution assay medium was transferred to all wells for a final assay volume of 250 *μ*L per well yielding a final concentration of 10% v/v Alamar blue. The plates were read against an excitation wavelength of 530 nm and an emission wavelength of 590 nm. It was recorded to determine whether any of the test compounds fluorescence at the emission wavelength thus interferes with the assay. Then plates were returned to incubator for 30 min and then fluorescence was read. The percent viability was determined as fluorescence counts in the presence of test compound as a percentage of that in the vehicle control. A compound is considered to be active only if it shows inhibition of 90%. The results were calculated in *μ*M concentrations and shown in Table 3.

### 3.4. *In Silico* Docking Studies

A crystallographic DNA gyrase with gap (PDB ID 4HYP, resolution 2.6 Å) was taken. This crystal structure was a tetramer with four endogenous binding sites for pyrrolopyrimidine molecules at ATP binding pockets. DNA gyrase B subunit was refined from the tetramer and water molecules were removed from all the structures by using Chimera (1.10.2). The energy minimizing process for molecule** 3a** was done using online server PRODRG. Molecular docking of compound** 3a** was performed with the DNA gyrase subunit B with crystallographic complexes 4HYP using Autodock 4.2.34. The rigid docking protocol was used, and the best binding mode of interaction was taken for MD simulation. Polar and aromatic hydrogens were added, and Gasteiger charges were computed by Autodock tool on each atom of the ligand. The AutoTors utility was used to define torsional degrees of freedom for the ligand. The grid box was centered in the macromolecule and the dimension of the grid was 80 × 72 × 80 Å with the spacing between the grid points at 0.514 Å in order to include the entire protein. Grid potential maps were calculated using the module AutoGrid 4.0. The Lamarckian genetic algorithm was used to perform docking simulation, with an initial population of 150 randomly placed individuals with a maximum number of 250000 energy evaluations, 150000 generations, mutation rate of 0.02, a crossover rate of 0.8, and an elitism value of 1, which were used. Then 100 docking runs were performed. Pseudo-Solis and Wets algorithm was used for local search method. Finally, the resulting docked conformations were clustered together on the basis of root-mean-square deviation (RMSD) tolerance of 2.0 Å and represented by most favorable free energy of binding. The resulting output files were viewed using discovery studio.

## 4. Conclusion

Design of quinoxaline containing sugar conjugates has been proven to be a beneficial attempt in search of new lead molecule to inhibit the growth of* Mycobacterium tuberculosis H*_*37*_ *Rv* and bacteria pathogens. It could serve as lead discovery strategy for further development due to their potential novel mechanism that binds to DNA gyrase. As continuation of this study, further using various monosaccharides with appropriate biosteric replacement strategy will certainly yield a lead molecule for MDR-TB and XDR-TB.

## Figures and Tables

**Table 1:** Physical data of the synthesized compounds.

Comp. code	% yield	Melting point	*R*_*f*_ value^a^	*λ* max in nm (ethanol)	log⁡*P* ^b^	Clog⁡*P* ^b^
**1**	64	212–13°C	0.715	247	−0.06	−0.292
**2**	62	>300°C	0.825	272	−0.03	−0.942
**3a** (glucose)	65	290–92°C	0.438	298	−1.89	−1.192
**3b** (mannose)	66	269–71°C	0.456	297	−1.89	−1.192
**3c** (maltose)	47	>300°C	0.628	286	−3.63	−2.935
**3d** (lactose)	44	>300°C	0.602	286	−3.63	−2.935
**3e** (ribose)	64	286–88°C	0.502	292	−1.36	−0.811
**3f** (xylose)	63	224–15°C	0.545	293	−1.36	−0.811

^a^TLC using silica gel GF254 and a mixture of chloroform and methanol (80 : 20% v/v). ^b^Values were calculated.

**Table 2:** Spectral characteristics of the synthesized compounds.

S. number	Comp. code	Molecular formula	Molecular weight	IR (cm^−1^)	^1^HNMR (*δ* ppm) (DMSO-d_6_/CDCl_3_)	^13^CNMR (*δ* ppm) (DMSO-d_6_/CDCl_3_)	ESI/MS
1	**1**	C_8_H_6_N_2_O_2_	162.14	3158 (NH), 1685 (C=O), 1612, 1500 (C=C stretching), 764 (ArC-H, out of plane)	11.90 (s, NH), 6.96–7.10, 7.11–7.14 (m, quinox).	155.6 (C=O), 125.3, 123.2, 115.4 (Ar C).	162 (M^+^)

2	**2**	C_8_H_8_N_4_O	176.17	3150 (NH weak), 1681 (C=O), 1560, 1500 (C=C), 759 (Ar C-H)	9.70 (s, NH), 7.89–7.81, 7.64–7.57 (m, quinox), 7.41 (s, NH), 6.8 (s, NH_2_)	155.6 (C=O), 148.3 (C=N) 142.1, 142.1, 136.4, 131.6, 130.9, 128.8, 128.1, 123.0, 122.4 (quinox).	176 (M^+^)

3	**3a**	C_15_H_18_N_4_O_6_	338.32	3450–3250 (OH stretch broad) 1683 (C=O), 1615 (C=N) 1072 (OH bend), 762 (ArC-H deform)	9.95 (s, NH), 8.26 (s, NH), 7.81–6.82 (m, Ar-H), 5.98 (s, NH), 4.91, 4.21 (s, anomeric CH), 3.92–2.95 (cpx, glucosyl)	160.2 (C=O), 148.7 (C=N) 142–122 (quinox) 98.2 (C^1^ CH–NH), 73.5, 72.4, 70.2, 69.2 (C^2^–C^5^), 63.1 (–CH_2 _OH).	339 (M^+1^)

4	**3b**	C_15_H_18_N_4_O_6_	338.32	3450–3250 (OH stretch broad) 1683 (C=O), 1615 (C=N) 1072 (OH bend), 762 (ArC-H deform)	9.95 (s, NH), 8.26 (s, NH), 7.81–6.82 (m, Ar-H), 5.98 (s, NH), 4.21 (s, anomeric CH), 3.92–2.95 (cpx, glucosyl)	160.2 (C=O), 148.7 (C=N) 142–122 (quinox) 98.2 (C^1^ CH–NH), 73.5, 72.4, 70.2, 69.2 (C^2^–C^5^), 63.1 (–CH_2 _OH).	339 (M^+1^)

5	**3c**	C_20_H_28_N_4_O_11_	500.45	3450–3250 (OH stretch broad) 1683 (C=O), 1615 (C=N) 1072 (OH bend), 762 (ArC-H deform)	10.22 (s, NH), 8.33 (s, NH), 7.96–6.90 (m, Ar-H), 6.11 (s, NH), 5.22 (s, anomeric CH), 4.23–3.19 (cpx, maltosyl)	161.1 (C=O), 148.9 (C=N) 143.1–121.5 (quinox) 100.2 (C^1^ CH–NH), 72–70 (maltosyl), 60.8 (–CH_2 _OH).	501 (M^+1^)

6	**3d**	C_20_H_28_N_4_O_11_	500.45	3450–3250 (OH stretch broad) 1683 (C=O), 1615 (C=N) 1072 (OH bend), 762 (ArC-H deform)	10.4 (s, NH), 8.33 (s, NH), 8.04–6.82 (m, Ar-H), 6.73 (s, NH), 5.58 (s, anomeric CH), 4.18–3.02 (cpx, lactosyl)	161.3 (C=O), 148.7 (C=N) 143.6–121.3 (quinox) 103.3 (C^1^ CH–NH), 71–76 (lactosyl), 61.2 (–CH_2 _OH).	501 (M^+1^)

7	**3e**	C_13_H_16_N_4_O_5_	308.29	3450–3250 (OH stretch broad) 1683 (C=O), 1615 (C=N) 1072 (OH bend), 762 (ArC-H deform)	10.13 (s, NH), 8.47 (s, NH), 7.92–6.85 (m, Ar-H), 6.24 (s, NH), 4.21 (s, anomeric CH), 2.23–4.37 (cpx, ribosyl)	161.8 (C=O), 150.5 (C=N) 144–123 (quinox) 96.5 (C^1^ CH–NH), 73.5, 71.8, 69.4 (C^2^–C^4^), 64.2 (–CH_2 _OH).	309 (M^+1^)

8	**3f**	C_13_H_16_N_4_O_5_	308.29	3450–3250 (OH stretch broad), 1683 (C=O), 1615 (C=N) 1072 (OH bend), 762 (ArC-H deform)	10.34 (s, NH), 8.65 (s, NH), 8.02–6.71 (m, Ar-H), 6.32 (s, NH), 4.78 (s, anomeric CH), 2.23–4.37 (cpx, Xylosyl)	162.1 (C=O), 150.0 (C=N) 143–121 (quinox), 97.5 (C^1^ CH–NH), 77.4, 75.1, 70.9 (C^2^–C^4^), 65.4 (–CH_2 _OH).	309 (M^+1^)

**Table 3:** Antibacterial and antitubercular activity of compounds synthesized (in *μ*M).

Comp. code	Antibacterial activity (MIC in *μ*M)					MIC against *Mycobacterium tuberculosis H*_37_ *Rv* (in *μ*M)
	SA	EC	PV	PA	ST	
**1**	29.6	14.8	29.6	29.6	>59.2	>59.2
**2**	13.6	3.6	13.6	13.6	>54.5	27.2
**3a**	14.2	7.1	14.2	14.2	>28.4	7.1
**3b**	>28.4	>28.4	>28.4	>28.4	>28.4	7.1
**3c**	>19.2	>19.2	>19.2	>19.2	>19.2	9.6
**3d**	>19.2	>19.2	>19.2	>19.2	>19.2	9.6
**3e**	2.07	2.07	2.07	2.07	7.8	0.65
**3f**	2.07	2.07	3.9	3.9	15.5	3.9
AMX	0.44	0.87	1.75	3.28	1.75	—
CIP	1.93	0.96	3.62	1.93	7.24	—
INH	—	—	—	—	—	0.145
Blank	—	—	—	—	—	—
